# Spinal Anaesthesia for Emergency Caesarean Section in a Morbid Obese Woman with Severe Preeclampsia

**DOI:** 10.1155/2012/586235

**Published:** 2012-10-14

**Authors:** Ebirim N. Longinus, Lagiri Benjamin, Buowari Yvonne Omiepirisa

**Affiliations:** Department of Anaesthesiology, University of Port Harcourt Teaching Hospital, Port Harcourt, 6173 Rivers State, Nigeria

## Abstract

*Background*. Morbid obesity in a pregnancy is a great challenge to medical practice especially when the patient requires caesarean section. *Case Summary*. A 38-year-old unbooked gravida 3 Para 2^+0^ weight 195 kg, height 1.7 m with a blood pressure of 210/160 mmhg had spinal anaesthesia for emergency caesarean section which was technically difficult for severe preeclampsia at 32-week gestation. She had poor wound healing and spent 18 days postoperatively on hospital admission. *Conclusion*. Morbid obesity is a challenge to both obstetric and anaesthetic practice. Antenatal care is necessary in reducing both maternal morbidity and mortality.

## 1. Introduction

Pre-eclampsia is a disorder that occurs in pregnancy after twenty weeks of gestation which manifests as hypertension and proteinuria, may progress to eclampsia and may regress following delivery [1–4]. Hypertension without proteinuria arising after twenty weeks gestation is referred to as pregnancy-induced hypertension [5]. Proteinuria is defined as at least 300 mg protein in a 24-hour urine collection or ++ dipstick 30 mg/dL in single urine sample [2]. The precise aetiology of preeclampsia is still unknown [1, 6], and multifactorial [7]. Preeclampsia is a disorder unique to human pregnancy [6], may involve the maternal cardiovascular, renal, coagulation, and hepatic system, and is associated with increased maternal and fatal morbidity and mortality [3, 8]. It occurs in 5–10% of all pregnancies [3, 5, 6]. Most definitions of hypertension in pregnancy are based on a diastolic blood pressure greater than 90 mmhg on two occasions or diastolic blood pressure greater than or equal to 110 mmhg on one occasion [4]. Four million women worldwide will develop preeclampsia annually and a further 100,000 will have eclampsia [7]. However, it remains a major cause of maternal and perinatal morbidity and mortality worldwide [6]. The incidence of preeclampsia varies according to the population studied and the criteria used for establishing the diagnosis [6]. Obesity is on the increase and carries increased morbidity and mortality in pregnancy [9]. Morbidly obese patients should be seen as high risk [9]. Obesity is a growing problem worldwide [10]. A subject is described as morbidly obese if the body mass index (BMI) is greater than 40 kg/m^2^ [11]. A case of a morbidly obese parturient that had a technically difficult spinal anaesthesia for emergency caesarean section for severe preeclampsia is presented. 

## 2. Case Presentation

 A 38-year-old unbooked gravida 3 Para 2^+0^ two alive woman with no formal education was booked at the antenatal clinic for elective caesarean section at 32 weeks for severe pre-eclampsia. Blood pressure at booking was 220/160 mmhg. Patient later went into spontaneous labour on the same day and developed cord prolapse for which she was booked for emergency caesarean section. On examination, she was in painful distress, anasarca, sacral oedema, bilateral pitting pedal oedema up to the knee joint, and periorbital oedema. Weight was 195 kg, height 1.7 m, pulse rate 100 beats per minute, and blood pressure 210/160 mmhg after receiving three doses of 5 mg diazepam, 10 mg hydralazine, and 25 mg magnesium sulphate. Auscultation of the chest was vesicular breath sounds. She was pale, anicteric, and febrile to touch. Urinalysis showed protein ++ and glucose +. Radom blood sugar was 6 mmol/dL. The temporomandibular joint was mobile with mallampati IV. Packed cell volume was 34%. An assessment of ASA IIIE with severe preeclampsia and prematurity in morbidly obese obstetric patient was made. The patient was counselled for surgery and informed consent obtained. Spinal anaesthesia was administered after several attempts with 2 mLs of hypobaric bupivacaine and L4, L5 interspace. The height of block was T4. She was given oxygen by facemask. During surgery, a male baby was extracted birth weight 2.1 kg, Apgar score [5, 7]. Blood pressure at the end of surgery was 200/160 mmhg. She spent several days in hospital because of poor wound healing and the blood pressure was persistently high despite administration of antihypertensive. She was discharged home 18 days postoperative due to industrial action by the hospital medical staff. 

## 3. Discussion

Both severe preeclampsia and eclampsia can seriously endanger the life of both mother and foetus and may account for up to 80% of maternal deaths in some parts of the developing world [3]. Preeclampsia and eclampsia remain among the most rewarding challenges in practice as a wide range of pathophysiological changes require an individualized approached to each case [12]. The incidence of preeclampsia is significantly increased in nulliparous women, in women with multiple gestations, in those with previous pre-eclampsia/eclampsia, and in women with underlying vascular or renal disease [6]. This patient was multiparous with a singleton pregnancy. Her two previous pregnancies were unsupervised, therefore, it cannot be deduced if there is a history of previous pre-eclampsia. She spent more days in hospital because she was morbidly obese and had pre-eclampsia. There are a number of potential problems relating to preeclampsia [13]. The choice of anaesthetic technique in severely pre-eclamptic women requiring caesarean section has been controversial for a number of years, but clinical experience has demonstrated the relative safety and value of well-managed incremental epidural anaesthesia [14]. The patient was given subarachnoid block (spinal anaesthesia) with hypobaric bupivacaine although it was technically difficult because of the patient's body size and subcutaneous fat. It was given in the sitting position. The benefits of spinal anaesthesia include rapid onset of reliable, high quality surgical anaesthesia, and avoidance of complications related to emergency general anaesthesia. The hazards of general anaesthesia in severe preeclampsia are well recognised. The anaesthetist often prefers the use of the sitting position in the obese patient since it is simpler to identify the midline and the obese patient prefers this position [15]. An obese patient poses a challenge to the anaesthetist. The obese patient is one of the anaesthetist nightmares as it carries a high morbidity and mortality [10]. In the obese parturient, there is increased oxygen consumption, abdominal weight that restricts the diaphragm and reduces chest wall compliance, difficult subarachnoid block, and wound infection [9]. The anaesthetic problems presented by the morbidly obese obstetric patient have risks and problems of a regional anaesthetic technique, particularly the technical difficulties. High spread can result in respiratory insufficiency and cardiovascular collapse [16]. The benefits of spinal anaesthesia include provision of a rapid, sense, and predictable block suitable for surgery while avoiding the risks of general anaesthesia. Caesarean section under general anaesthesia in severe preeclampsia is a high-risk procedure. The factors, which make general anaesthesia in preeclampsia particularly hazardous, include the increased risk of difficult airway and intubation and marked pressor response at laryngoscopy, intubation and extubation resulting in dangerous surges in blood pressure. There is a significant risk of intracranial haemorrhage secondary to uncontrolled severe hypertension at induction of general anaesthesia [4]. Generally, the decision when to deliver remains a clinical one based on gestation and foetal and maternal condition with the mother always taking priority if there is significant increase in maternal risk [7]. The benefits of antenatal care cannot be over emphasized most especially in the reduction of maternal and perinatal morbidity and mortality [17].

## 4. Conclusion

Morbid obesity is a challenge to anaesthetic and obstetric practice as its problems of transportation of the patient from the ward to the operating room, wound healing, and difficulties in establishing regional blocks because of difficulties in identifying landmarks. Longer needles are required, which are not readily available in developing countries like Nigeria. 
